# Dataset on fire resistance analysis of FRP-strengthened concrete beams

**DOI:** 10.1016/j.dib.2024.110031

**Published:** 2024-01-05

**Authors:** P.P. Bhatt, V.K.R. Kodur, M.Z. Naser

**Affiliations:** a Walter P. Moore, Kansas City, MO, USA; b Department of Civil and Environmental Engineering**,** Michigan State University, MI, USA; c School of Civil and Environmental Engineering & Earth Sciences, Artificial Intelligence Research Institute for Science and Engineering, Clemson University, SC 29634, USA

**Keywords:** Fiber reinforced polymer (FRP), Fire resistance, Concrete beams, FRP-strengthening, Fire exposure, Machine learning, Artificial intelligence

## Abstract

Machine learning (ML) has emerged as an efficient and feasible technique for tackling engineering problems. Despite the numerous advantages, the implementation of ML for evaluating the fire resistance of structural members is relatively scarce, primarily due to the lack of a reliable database with a substantial number of data points. To address this knowledge gap, this paper presents a comprehensive database on the fire performance of fiber reinforced polymer (FRP) strengthened reinforced concrete (RC) beams. The database comprises over 21,000 experimental and numerical data points with varying parameters, including various geometric dimensions, FRP-strengthening levels, steel reinforcement ratio, insulation thickness and configuration, material properties, and applied load levels. The database can be implemented to train ML algorithms for developing autonomous models for predicting the fire resistance of FRP-strengthened concrete beams with varying parameters.

Specifications Table

**Table:** 

Subject	Civil and Structural Engineering.
Specific subject area	Evaluation of fire resistance of fiber reinforced polymer (FRP) strengthened reinforced concrete (RC) beams
Data format	Analyzed
Type of data	Table
Data collection	The data presented in this study was collected using three different approaches, as listed below: 1) Fire resistance tests were conducted on FRP-strengthened RC beams and slabs at Michigan State University 2) Fire test data on FRP-strengthened concrete beams available in open literature was collected and compiled. 3) Numerical model developed and validated by Kodur and Bhatt was applied to conduct fire resistance analysis on a series of FRP-strengthened RC beams with different geometrical dimensions, insulation configuration, applied loading, and fire duration.
Data source location	Institution: Michigan State University City/Town/Region: East Lansing, Michigan Country: United States of America
Data accessibility	Repository name: Fire Resistance of FRP-Strengthened Beams (**Version 6**) Data identification number: DOI: 10.17632/3c2szhbdn5.6 Direct URL to data: https://data.mendeley.com/datasets/3c2szhbdn5/6 Instructions for accessing these data: Directly accessible through the above link. Please follow the following steps to access the excel file: **Step 1-** Please expand the files drop down menu on repository webpage by selecting option “**Version 6**” available on right hand side of Mendeley Data repository webpage. **Step 2-**Click on the excel file named “Dataset_FireResistanceofFRP-StrengthenedBeams_PB_Ver6.0.xlsx”.
Related research article	• Bhatt, P. P. (2021). Fire performance of FRP-strengthened concrete flexural members. doi:10.25335/d4ef-qv21 • Bhatt, P. P., Kodur, V. K. R., Shakya, A. M., Alkrdaji, T. (2020). “Performance of Insulated FRP-strengthened Concrete Flexural Members under Fire Conditions.” *Frontiers of Structural and Civil Engineering,* 15, 177–193. 10.1007/s11709-021-0714-z • Kodur, V. K. R., and Bhatt, P. P. (2018). “A Numerical Approach for Modeling Response of Fiber Reinforced Polymer Strengthened Concrete Slabs Exposed to Fire”, *Composite Structures,* 187, 226–240. 10.1016/j.compstruct.2017.12.051

## 1 Value of Data

The value of this database stems from the nature and variety of the generated data. for example,

• To date, this is the largest database, with over 21,000 experimental and numerical data points.
• The database includes various parameters, such as the geometric dimensions of beams, FRP-strengthening levels, steel reinforcement ratio, insulation thickness and configuration, material properties, and applied load levels.
• This database can be used to train machine learning algorithms or conduct statistical analyses.
• The data can also be used to update provisions and guidelines in building codes and standards.

## 2 Data Description

The database [2] is provided in a Microsoft Excel file (*Dataset_FireResistanceofFRP-StrengthenedBeams_PB.xlsx*), consisting of three spreadsheets titled as, “01_FireTestData”, “02_NumericalModelData”, and “03_Notations”, respectively. The first two spreadsheets, i.e., “01_ FireTestData” and “02_NumericalModelData”, comprises of the datapoints pertaining to fire resistance of FRP-strengthened RC beams, while the third spreadsheet, i.e., “03_Notations” provides the definition of the notations used as column headers in the first two sheets.

The fire resistance data presented in sheets “01_FireTestData”and “02_NumericalModelData”, is referred to as Dataset-E and Dataset-N, respectively. Dataset-E comprises of 49 data points which are compiled using the fire test data on FRP-strengthened concrete beams reported in the literature [3], [4], [5], [6], [7], [8], [9], [10], [11], [12]. Whereas Dataset-N comprises of 21,384 data points generated numerically by applying the numerical model presented in Kodur and Bhatt [1], on a series of FRP-strengthened RC beams with varying parameters. Each datapoint in both the datasets is identified using a unique label known as “Beam Name (BN)”, defined in column A of the spreadsheets. Following the identification, 20 different parameters are provided in columns B to U of the spreadsheets. These parameters can be grouped into four different categories, namely geometrical parameters, material property parameters, loading parameters, and fire resistance parameters.

The geometrical parameters comprise of length of beam (*L*), area of concrete (*A_c_*), cover to steel reinforcement (*C_c_*), total area of tensile steel reinforcement (*A_s_*), area of FRP (*A_f_*), the thickness of insulation (*t_ins_*), and depth of insulation on sides of beams (*h_i_*), and are defined in columns B, C, D, E, F, G, and H respectively. Similarly, the material property parameters comprise of compressive strength of concrete (*f_c_*), yield strength of steel rebars (*f_y_*), elastic modulus of FRP (*E_s_*), ultimate tensile strength of FRP (*f_u_*), elastic modulus of steel reinforcement (*E_frp_*), glass transition temperature of FRP (*T_g_*), thermal conductivity of insulation (*k_ins_*), and specific heat capacity of insulation (ρ*_ins_ c_ins_*), which are defined in columns I, J, K, L, M, N, O, and P, respectively. In the current database, the Dataset-E (spreadsheet “01_FireTestData”), only the beams tested under ASTM E119, or ISO 834 standard fire exposures were included. Similarly, all the beams in Dataset-N (spreadsheet “02_NumericalModelData”) were analyzed under standard fire exposure. Hence, there is no variation in thermal loading parameters of the beams. Therefore, only the structural loading parameters namely, total applied loading (Ld) and load ratio (LR) are provided loading parameters group and are defined in columns Q and R, respectively. The fourth group, i.e., fire resistance group provides information pertaining to failure of the beam (F/EF), failure time of the beam (FR), and mid-span deflection (δ_f_) at failure or the end of fire exposure in columns S, T, and U, respectively. The failure of beam defined in column S provides a binary value, F/EF = 0, when the beam has failed, and F/EF = 1, when the beam did not fail but the fire exposure ended.

To generate the Dataset-N, the parameters in the first three categories, i.e., geometrical parameters, material property parameters, loading parameters were varied over a wide range to incorporate all the possible values used in practical field implementation. These range of values were determined through consultation with construction industry personnels and based on the experience of the authors. For instance, the range for geometrical and material properties of concrete and steel reinforcement was determined based on the consideration that FRP strengthening is typically applied to aging or deficient structures which are constructed with normal strength concrete and has two the three twisted high strength steel rebars in tension zone. Whereas the range for geometrical configuration and material properties of FRP sheets and fire insulation was determined based on the commonly available materials in the market. The range of loading parameters, i.e., applied structural loading and fire exposure duration was determined to account for building code provisions, i.e., a strengthened/un-strengthened reinforced concrete beam should attain one to three hours of fire resistance under service load conditions. The specific range of values for different parameters implemented to generate the dataset are summarized in Table 1.

**Table 1:** Range of values considered for the parameters used of generating the Dataset -N.

Parameter category	Description	Minimum	Maximum
Geometrical configuration parameters	Length of beam (m)	1	6
	Width of concrete beam (mm)	100 -	350
	Depth of concrete beam (mm)	120	450
	Concrete cover (mm)	10	38
	Diameter of tension steel rebar (mm)	10	25
	Number of tension steel rebar (#)	2	3
	Width of FRP sheet (mm)	10	320
	Thickness of FRP sheet (mm)	0.11	2.00
	Thickness of insulation (mm)	0	38
	Depth of insulation on sides (mm)	0	115
Material property parameters	Compressive strength of concrete (MPa)	25	45
	Yield strength of steel (MPa)	414	460
	Elastic modulus of steel reinforcement (MPa)	200,000	210,000
	Tensile strength of FRP (MPa)	900	4900
	Elastic modulus of FRP reinforcement (MPa)	66,000	255,530
	Glass transition temperature of polymer (°C)	60	80
	Thermal conductivity of insulation (W/mK)	0.037	0.228
	Specific heat capacity of insulation (J/°C m^3^)	240,000	1,031,000
Loading parameters	Total load applied on beams (kN)	3	220
	Applied load ratio (%)	15	75
	Fire exposure time (minutes)	60	300

The above-described database or a subset of it can be utilized to train different machine learning algorithms for predicting different response parameters such as, fire resistance, deflection at failure, etc., for a fire exposed FRP-strengthened RC beams. For instance, a subset of Dataset-E (spreadsheet “01_FireTestData”) was utilized by Bhatt and Sharma [13] to train a deep neural network model for predicting fire resistance of FRP-strengthened RC beams. This subset of the Dataset-E utilized by Bhatt and Sharma [13] for training the model is shown in Table 2.

**Table 2:** Summary of Dataset-E based on fire tests reported in literature.

*Beam Name*	*L*	*A_c_*	*A_s_*	*A_f_*	*C_c_*	*t_ins_*	*h_i_*	*f_c_*	*f_y_*	*f_u_*	*T_g_*	*k_ins_*	*ρ_ins_ c_ins_*	*Ld*	*FR*	Reference No
	(m)	(mm^2^)			(mm)			( MPa)			°C	(W/mK)	(J/kg °C m^3^)	kN	(min)	#
B1	3	60,000	402	0	25	0	0	48	591	0	0	0	0	61.2	90	
B2	3	60,000	402	0	25	0	0	46	591	0	0	0	0	61.2	90	
B3	3	60,000	402	120	25	25	0	44	591	2800	52	0.175	730,800	81.2	76	
B4	3	60,000	402	120	25	40	80	47	591	2800	52	0.175	730,800	81.2	90	
B5	3	60,000	402	120	25	25	80	45	591	2800	52	0.175	730,800	81.2	92	
B6	3	60,000	402	120	25	0	0	46	591	2800	52	0.175	730,800	81.2	76	
B7	3	60,000	402	120	25	25	0	48	591	2800	52	0.175	730,800	81.2	90	
B8	3	60,000	402	120	25	25	0	44	591	2800	52	0.125	735,000	81.2	91	
B9	6	100,000	402	67	20	40	500	23	375	4030	73	0.06	193,550	121	84	
B10	3.7	103,124	851	0	38	0	0	52	450	0	0	0	0	100	180	
B11	3.7	103,124	851	460	38	25	100	52	450	986	82	0.116	245,700	140.	180	
B12	1.5	12,000	57	0	10	0	0	26	542	0	0	0	0	10.2	69	
B13	1.5	12,000	57	60	10	0	0	26	542	2742	55	0	0	16.3	60	
B14	1.5	12,000	57	60	10	25	0	26	542	2742	55	0.058	326,325	16.3	90	
B15	1.5	12,000	57	60	10	25	0	26	542	2742	55	0.164	619,440	16.3	89	
B16	1.5	12,000	57	60	10	40	0	26	542	2742	55	0.058	326,325	16.3	208	
B17	1.5	12,000	57	60	10	40	0	26	542	2742	55	0.164	619,440	16.3	181	
B18	1.26	12,000	57	0	15	0	0	37	546	0	0	0	0	7.2	50	
B19	1.26	12,000	57	28	15	0	0	37	546	2076	47	0	0	23.4	15	
B20	1.26	12,000	57	28	15	0	0	37	546	2076	47	0.09	366,750	23.4	31	
B21	1.26	12,000	57	28	15	25	0	37	546	2076	47	0.09	366,750	23.4	32	
B22	1.26	12,000	57	28	15	25	0	37	546	2076	47	0.09	366,750	23.4	50	
B23	1.26	12,000	57	28	15	25	0	37	546	2076	47	0.09	366,750	23.4	75	
B24	1.26	12,000	57	28	15	50	0	37	546	2076	47	0.09	366,750	23.4	80	
B25	1.26	12,000	57	28	15	25	0	37	546	2076	85	0.09	366,750	23.4	74	
B26	1.26	12,000	57	28	15	0	0	37	546	2076	47	0	0	23.4	12	
B27	1.26	12,000	57	28	15	25	0	37	546	2076	47	0.09	366,750	23.4	61	
B28	4.7	90,000	402	33	20	50	450	31	372	4030	73	0.12	500,000	80.0	149	
B29	4.7	90,000	402	33	20	20	200	31	372	4030	73	0.12	500,000	80.0	117	
B30	5.2	100,000	943	33	20	40	500	31	372	4030	73	0.061	185,000	132	120	
B31	5.2	100,000	943	33	20	1.5	500	31	372	4030	73	0.06	480,000	132	123	
B32	4.4	100,000	760	0	25	0	0	40	364	0	0	0	0	76.0	129	
B33	4.4	100,000	760	42	25	10	80	40	364	3455	85	0.126	518,000	102	199	
B34	3	45,000	157	0	19	0	0	30	500	0	0	0	0	48.0	77	
B35	3	45,000	157	60	19	20	300	30	500	2742	75	0.13	594,000	48.0	100	
B36	3	45,000	157	60	19	35	300	30	500	2742	75	0.13	594,000	48.0	108	
B37	3	45,000	157	60	19	50	300	30	500	2742	75	0.13	594,000	48.0	127	
B38	3	45,000	157	60	19	20	300	30	500	2742	75	0.67	1,320,000	48.0	92	
B39	3	45,000	157	60	19	35	300	30	500	2742	75	0.67	1,320,000	48.0	114	
B40	3	45,000	157	60	19	50	300	30	500	2742	75	0.67	1,320,000	48.0	104	
B41	3	45,000	157	60	19	20	300	30	500	2742	75	0.058	427,500	48.0	86	
B42	3	45,000	157	60	19	35	300	30	500	2742	75	0.058	427,500	48.0	128	
B43	3	45,000	157	60	19	50	300	30	500	2742	75	0.058	427,500	48.0	128	
B44	3.66	60,800	339	77	19	19	152	48	500	1172	80	0.156	510,000	21.0	180	
B45	3.66	60,800	339	77	19	25	152	48	500	1172	80	0.156	510,000	26.0	180	
B45	3.66	125,730	603	102	38	32	152	46	460	1172	82	0.156	510,000	97	240	
B46	3.66	125,730	898	102	38	19	152	46	450	1172	82	0.156	510,000	128	240	
B47	3.66	125,730	603	173	38	0	0	42	440	1034	82	0	510,000	98.	180	
B48	3.66	125,730	603	173	38	25	75	42	440	1034	82	0.156	510,000	98.0	180	
B49	3.66	125,730	603	173	38	19	112	42	440	1034	82	0.156	510,000	116	176	

*Note:* L: length of beam (m); Ac: Area of concrete (mm2); As: Area of steel (mm2); Af: Area of FRP (mm2); Cc: concrete cover (mm); tins: thickness of insulation at midspan (mm); ***h_i_***: depth of insulation on sides (mm); ***f_c_***: compressive strength of concrete (MPa); ***f_y_***: yield strength of steel (MPa); ***f_u_***: tensile strength of FRP (MPa); ***T_g_***: glass transition temperature of polymer (°C); ***k_ins_***: thermal conductivity of insulation (W/mK); ***ρ_ins_ c_ins_***: Specific heat capacity of insulation (J/°C m^3^); ***Ld***: total load applied on beams (kN); ***FR***: fire resistance time (minutes).

## 3 Experimental Design Materials and Methods

The experimental program consisted of a fire resistance test on two RC T-beams designated as TB1 and TB2. Details of the specimens and test procedure are described below.

### 3.1 Specimen details

**Fig. 1:**
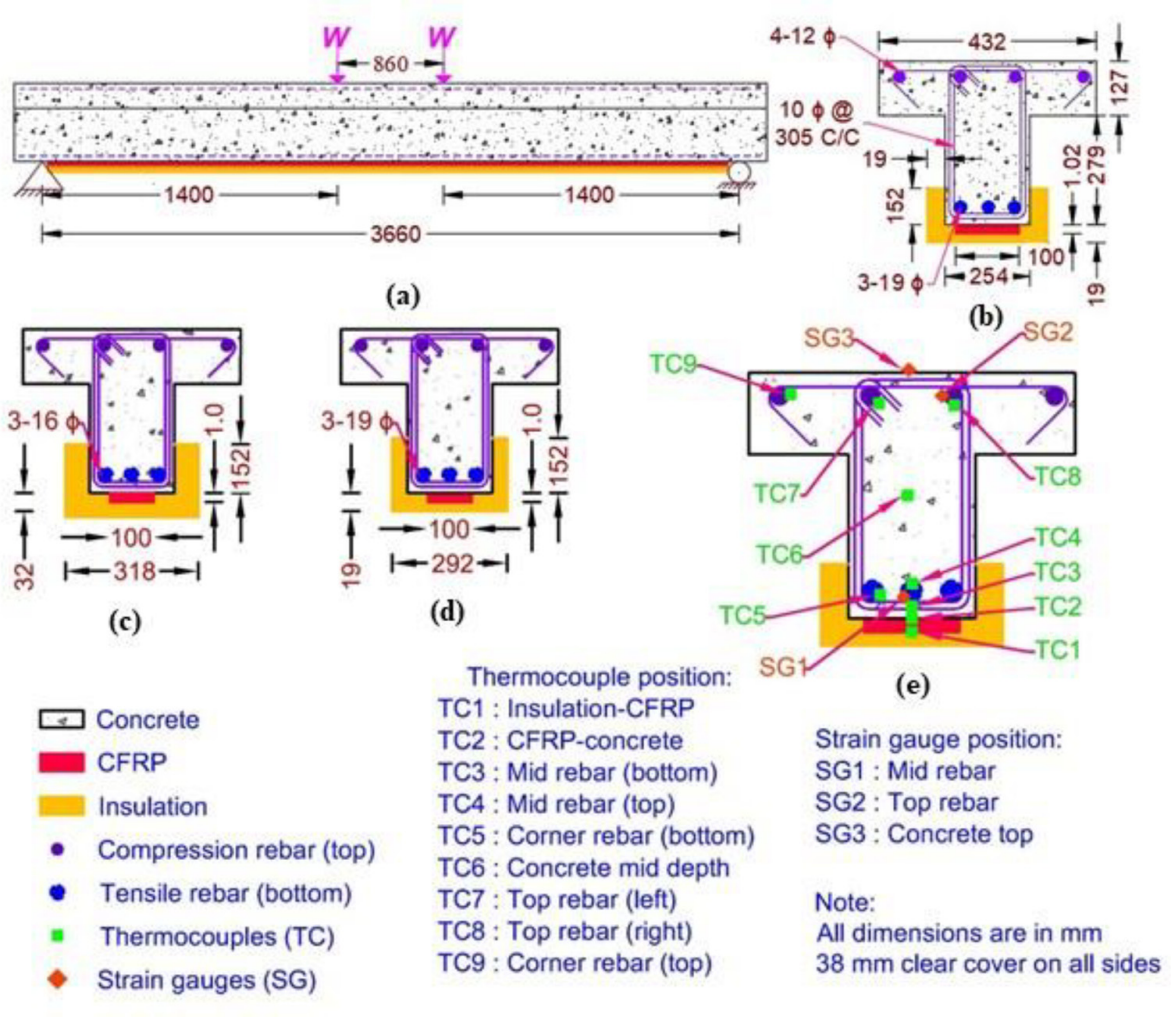
Geometrical details of tested specimens: (a) elevation of a typical T-beam (b) cross-section of a typical T-beam (c) cross-section of beam TB1 (d) cross-section of beam TB2 (e) location of thermocouple and strain gauges in cross-section of beams TB1 and TB2.

Fig. 1 shows the elevation and cross-section details of the beams. Both the beams had a span length of 3960 mm long with a clear span length of 3660 mm and cross-section dimensions of 432 mm × 127 mm and 254 mm × 279 mm (width × depth) for the flange and the web, respectively, as shown in Fig. 1 (a-b). The beams TB1 and TB2 had 3–16 mm ϕ and 3–19 mm ϕ rebars (Grade 60), respectively, as the main flexural reinforcement and 4–12 mm ϕ rebars as the compressive reinforcement. These reinforcing bars had a clear cover of 38 mm. Additionally, in both the beams, 10 mm ϕ stirrups spaced at 152 mm c/c were provided as shear reinforcement, and 10 mm ϕ rebars spaced at 305 mm c/c were provided as transverse reinforcement in the flanges. Both the beams were fabricated at the Civil Infrastructure Laboratory (CIL) of Michigan State University (MSU) using the same concrete mix, which comprised of type-I Portland cement, carbonate based coarse aggregate, and silica-based fine aggregates and yielded an average cylindrical compressive strength of 43 MPa on 28th day, which increased to 46 MPa on test day.

The fabricated specimens were stored at room temperature conditions for six months and then flexurally strengthened by applying a 1 mm thick CFRP laminate (commercial designation V-Wrap C200HM) in their tension zone (soffit) for 3660 mm. Both the beams TB1 and TB2 were strengthened with a 100 mm wide CFRP laminate which increased their moment capacity by 23% and 33%, respectively. The CFRP laminate used for strengthening had a design ultimate tensile strength, design tensile modulus, and a rupture strain of 1172 MPa, 96.5 GPa, and 1.1%, respectively. Whereas the epoxy adhesive (commercial designation V-Wrap 700S) used for bonding the CFRP laminate had the tensile strength, tensile modulus, and rupture strain of 45 MPa, 2.07 GPa, and 5.5%, respectively, and had a glass transition temperature of 80 °C evaluated by the manufacturer as per ASTM-D4065 specifications.

The strengthened beams were cured for 24 h and then spray-applied with cementitious fire insulation material (V-Wrap FPS) on the soffit and sides up to 152 mm throughout the span. The cementitious mixture comprised vermiculite, gypsum, and Portland cement along with a few other additives and had a density of 425 kg/m^3^ with thermal conductivity and specific heat of 0.156 W/m-K and 1888 J/kg-K, respectively. The insulation layer thickness applied on beam specimens TB1 and TB2 was 32 mm and 19 mm, respectively, as shown in Fig. 1 (c-d). Special attention was paid to maintaining uniform insulation thickness throughout the beams and slabs. Insulation thickness was measured at several places along the beam length to ensure that insulation thickness was uniform within a tolerance of ± 3 mm (1/8 inch)*.*

The beams were instrumented with Type-K thermocouples at two different sections along the span length to measure the temperature rise at various depths in concrete, on steel rebars, and at the interfaces of CFRP-concrete and insulation-CFPR, as shown in Fig. 1(e). Additionally, standard temperature strain gauges were installed on the top and bottom rebars at the mid-span section of the beams to monitor strain in reinforcement. Also, two linear variable differential transducers were installed at the mid-span section on the unexposed surface of the beams and slabs to record the mid-span deflection during the fire exposure.

### 3.2 Test setup and procedure

**Fig. 2:**
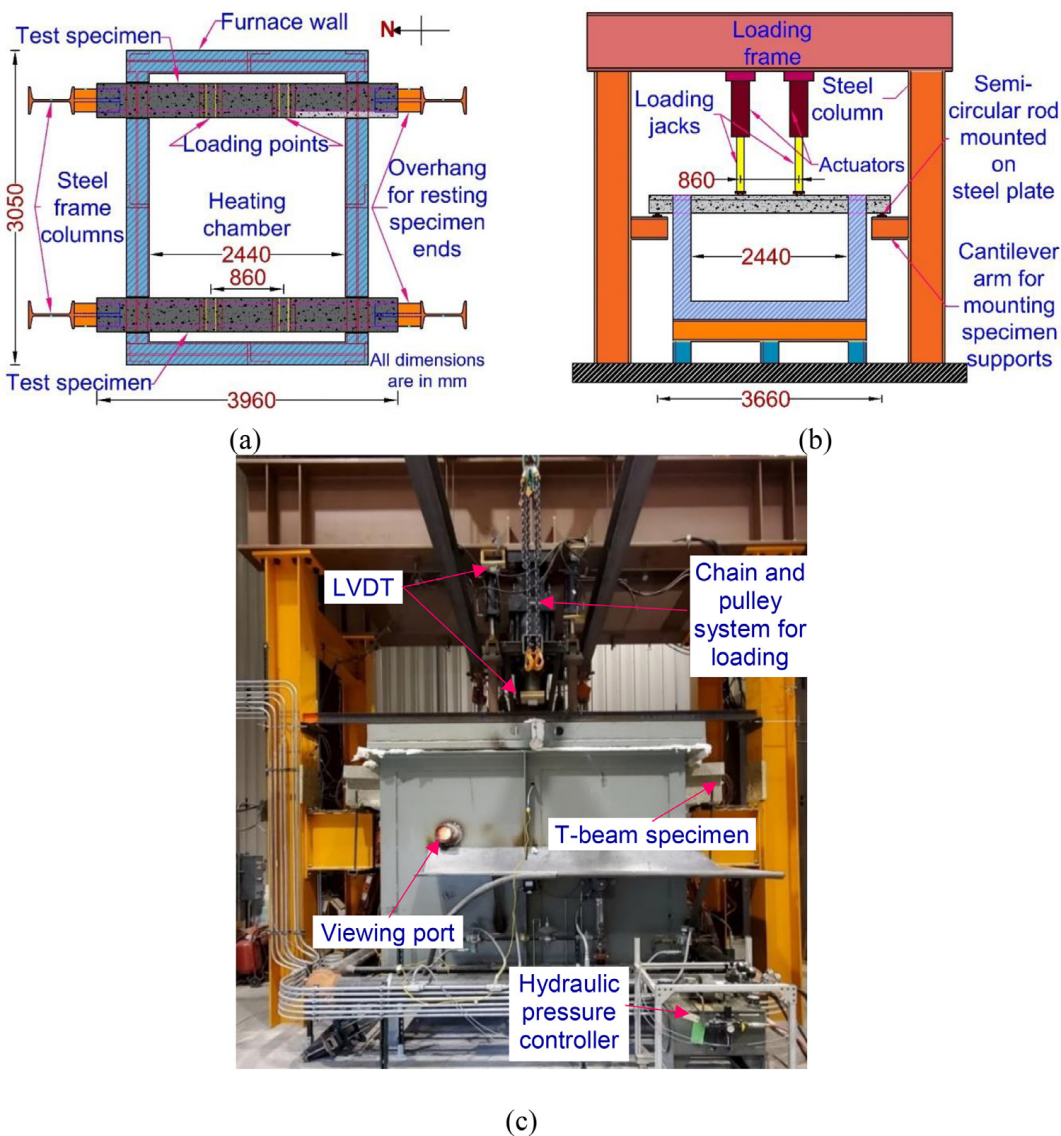
Structural fire test furnace at MSU's Civil Infrastructure Laboratory with specimen loaded: (a) plan view (b) front view (EW elevation) (c) T-beams in the furnace.

The two T-beams (TB1 and TB2) were tested simultaneously in the fire test conducted at the structural fire testing facility at Michigan State University. The test facility comprises a loading steel frame supported on four steel columns and a fire chamber 3.05 m long, 2.44 m wide, and 1.68 m high. The fire chamber is equipped with six propane burners and six type-K internal thermocouples to provide thermal energy and monitor the furnace temperature during a fire test. The test furnace can simultaneously apply both structural and thermal (heating) loading conditions. The fire furnace and the test setup are shown in Fig. 2, wherein Fig. 2 (a and b) shows the schematic layout of the furnace in plan and front view (east-west elevation), respectively, while the fire furnace, together with T-beams mounted in it, is shown in Fig. 2(c).

The test was carried out in two steps; in the first step, a pre-determined structural load was applied on the beams until the steady state condition, i.e., no increase in deflection with time, was reached. After this, the specimens were exposed to ASTM E119 standard fire on three surfaces, i.e., two sides and the bottom surface, while the structural loading was maintained constant.

During the fire test, both the beams had simply supported end conditions, with an unsupported length of 3.6 m, of which 2.44 m was exposed to heating in the furnace. The structural loading was applied in the form of two concentrated loads placed at 1.4 m from the end supports. A total load of 97 kN and 128 kN was applied on beams TB1 and TB2, respectively, representing about 66% and 63% of ultimate strengthened capacity at room temperature.

The fire exposure on the beams (TB1 and TB2) was continued for 4 h, during which the deflection at the mid-span, strains, and temperatures at various locations was monitored and recorded using state of the art data acquisition system. Data generated from the fire test was utilized to evaluate the fire performance of CFRP-strengthened RC beams.

## 4 Results

**Fig. 3:**
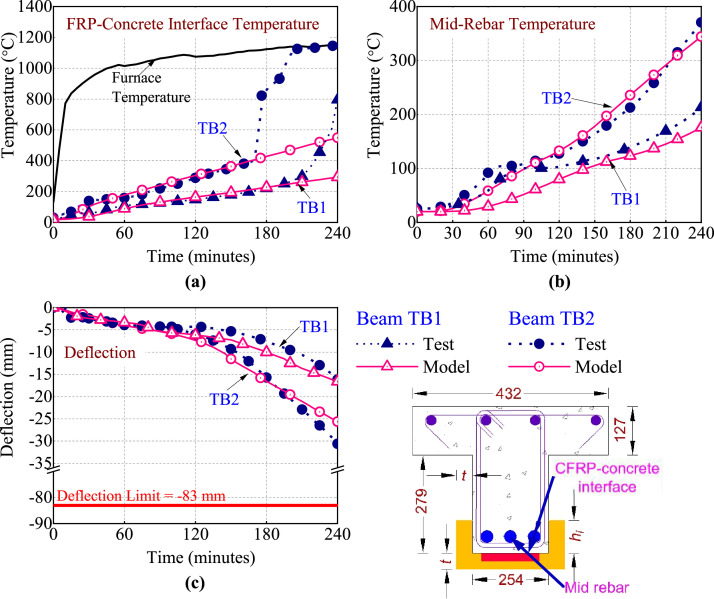
Thermal and structural response of beams TB1 and TB2 measured in fire tests and predicted by numerical model: (a) FRP-Concrete interface temperature (b) Mid-rebar temperature (c) mid-span deflection.

The fire performance is evaluated in terms of thermal response and structural response of the beams. The thermal response of beams TB1 and TB2 is evaluated by plotting the temperature rise at FRP-concrete interface and steel rebars in the middle of the beams cross-section, as a function of fire exposure time in Fig. 3 (a, and b), respectively. Additionally, the furnace gas temperature measured during the test is also shown in Fig. 3(a). It can be from Fig. 3 (a-b) that due to larger thickness of insulation on beam TB1, the temperature rise at any point in beam TB1 is slower than the temperature rise at corresponding point beam TB2. The interface temperature in beams TB1 and TB2 (Fig. 3(a)) exceeds the adhesive *T_g_* (80 °C) after 44 and 18 min of fire exposure, respectively. Further, the interface temperature in both the beams increases sharply and approaches furnace temperature at about 220 and 170 min of fire exposure, respectively, which indicates localized delamination of the fire insulation. The mid steel rebar temperature of beams TB1 and TB2 increases slowly in the initial stages of fire exposure and reaches a concrete water evaporation plateau at about 75 and 60 min, respectively. The steel rebar temperature in both the beams continue to increase gradually beyond the plateau and remains below 400 °C the end of 240 min of fire exposure, indicating minimal strength loss in rebars.

The structural response of beams TB1 and TB2 is evaluated by plotting the mid-span deflection as a function of fire exposure time, as shown in Fig. 3(c). Deflection of beams TB1 and TB2 progresses gradually at the same rate and TB2 increases gradually from the early stages to about 150 and 120 min of fire exposure, respectively. After this point, the deflection in beams TB1 and TB2 starts increasing at a relatively faster pace. This can be attributed to the reduction in the strength and stiffness of CFRP sheet with increase in temperature as well as to the loss of bond between CFRP and concrete. Although the FRP-concrete interface temperature of beams TB1 and TB2 exceeds the adhesive *T_g_* at 44 min and 18 min of fire exposure, respectively, the increase in deflection of beams commences at a much later stage. This can be attributed to the fact that attainment of *T_g_* at the interface does not lead to an immediate loss of bond between FRP-concrete, and hence, FRP continues to contribute to the strength and stiffness of beams at temperature much higher than adhesive *T_g_* [12]. The maximum deflection observed at the end of fire exposure, in beams TB1 and TB2 was 16 mm and 30 mm, respectively. Thus, both the beams attained very low deflection and sustained applied load without failure until the end of fire exposure. Therefore, the beams (TB1 and TB2) achieved 4 h of fire resistance, respectively. as per ASTM E119 [14] limiting criterion. For a detailed evaluation of the fire performance of the CFRP-strengthened beams the reader is encouraged to refer the Bhatt et al. [11] and Bhatt [12] documents.

## 5 Numerical model

A macroscopic finite element based numerical model developed using FORTRAN programming is applied to develop the database on fire performance of FRP-strengthened RC beams. This model initially developed by Kodur and Ahmed [15] for evaluating fire response of FRP-strengthened RC beams later modified by Kodur and Bhatt [1] to evaluate the fire resistance of FRP-strengthened RC beams and slabs. The model utilizes a member level approach and accounts for temperature induced degradation of material properties, including bond, as well as different failure limit states to evaluate the response of strengthened flexural members from loading in a linear elastic range to collapse under fire. The details pertaining to the general procedure, failure criteria and material properties implemented in the model as well as the validation of model are described in this section.

### 5.1 Numerical procedure

**Fig. 4:**
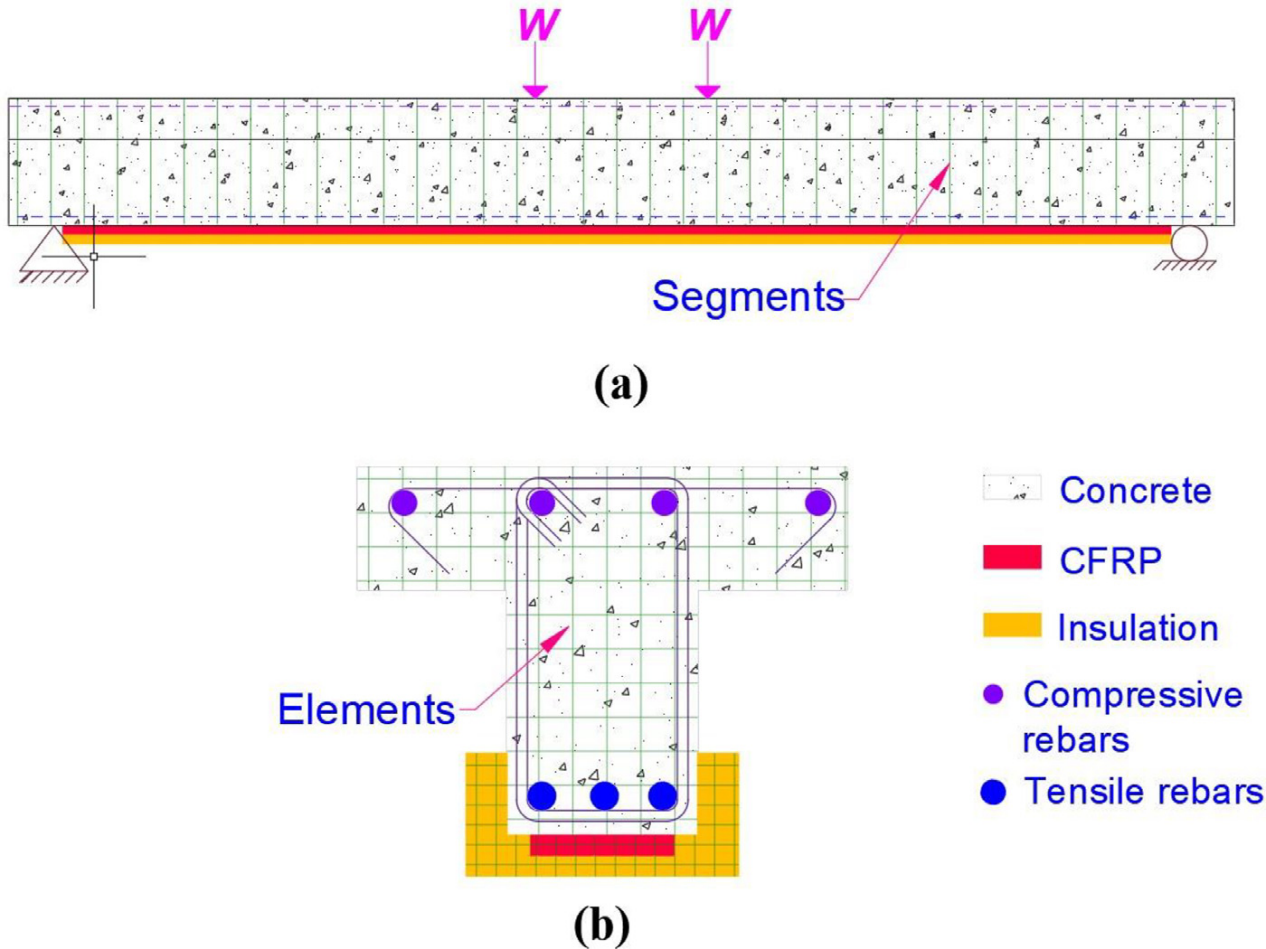
Discretization of the beam into segments and elements: (a) discretization of length in segments (b) discretization of cross-section into elements.

**Fig. 5:**
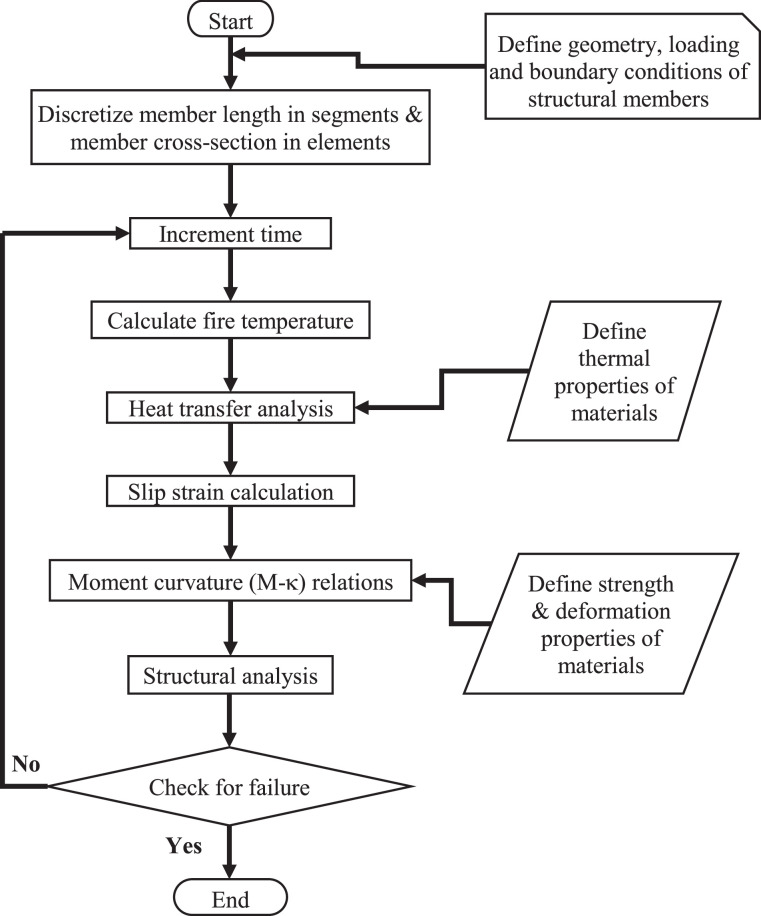
Flowchart illustrating the steps followed in macroscopic finite element model for evaluating the response of CFRP-strengthened RC beams.

A CFRP-strengthened RC beam typically comprises of a RC beam with CFRP-strengthening system applied at the bottom surface and with optional fire insulation provided at the bottom and two side surfaces, as shown in Fig. 4. During fire exposure, the two sides and bottom surface of FRP strengthened RC beam are exposed to heat of fire, while ambient conditions prevail on top of the beam. To evaluate the fire resistance of such a CFRP-strengthened RC beam member, a four-step numerical procedure is implemented in the model. These steps include, (i) discretization of beam, (ii) computation of fire temperature computation, (iii) thermal analysis, (iv) structural analysis. The execution sequence of these four steps is illustrated through a flowchart, shown in Fig. 5 and are explained here.

(i) Discretization

At the start of the fire resistance analysis the strengthened beam member is discretized into 20–30 segments along its length as shown in as shown in Fig. 4(a), while the cross-section of each segment is discretized into a mesh of rectangular elements as shown in Fig. 4(b). A coarser mesh size is adopted for discretizing the concrete cross-section typically 10 mm × 10 mm. Whereas a finer mesh size (typically, 1 mm × 1 mm) is adopted for discretizing the CFRP and insulation material, as they are in close proximity of the heat source (fire).

(ii) Computation of fire temperature

Following the discretization, fire exposure condition is applied on the bottom and two sides of the beam in small incrementing time steps of 30 s each. At each time-step the fire temperature is computed through a specific time-temperature relation, as per standard fire curve (ASTM E119 or ISO 834) or any given design fire scenario. After applying the fire temperature, a sequential coupled thermos-mechanical analysis is carried out at each time step.

(iii) Thermal analysis

In the thermal analysis, the temperature distribution within the cross-section of each segment is evaluated through a heat transfer analysis, wherein temperature dependent thermal properties of constituent materials, namely, concrete, steel rebars, FRP and insulation are taken into account. The temperature rise in the section is computed by establishing a heat balance at each element, and the temperature distribution in the cross-section is assumed to be uniform along the entire length of the segment. Further, the temperature at the center of the element is computed by taking the mean of temperatures at the nodes of rectangular elements, which is then provided as an input to the structural analysis.

(iv) Structural analysis

In the structural analysis, the moment capacity and deflection of the beam are evaluated. The structural analysis starts by computing strains, at each segment, resulting from relative slip between SRP and concrete, due to degradation of the interfacial bond. Following this, the moment-curvature (M-κ) relationships for each segment are generated taking into account temperature dependent degradation in mechanical properties of constituent materials. These M-κ relations for each segment are generated through an iterative procedure, wherein the curvature corresponding to the strain in the topmost fiber of concrete is iterated until force equilibrium and strain compatibility conditions in each segment are satisfied, within a specified numerical (convergence) tolerance. The maximum value of the moment in the M-κ relations, determines the moment capacity of the beam. Following the sectional analysis, the secant stiffness matrix of each segment is computed using the M- κ relations generated for each segment. These secant stiffness matrices of each segment are then utilized in a stiffness analysis, to evaluate deflection of the beam. The moment capacity and deflection of the beam are then compared with limits states (described below) to evaluate failure of the beam.

### 5.2 Failure criteria

Strength and serviceability failure criteria specified in ASTM E119 [14] are implemented in the model to evaluate the fire resistance of the FRP-strengthened beam. As per the strength criterion, the beam is considered to have failed when the bending moment due to applied loading exceeds the moment capacity of the section. Whereas as per the serviceability criterion, the beam is deemed to have failed when the mid-span deflection of the beam exceeds *L*^2^/400*d*, and the rate of deflection exceeds *L*^2^/9000d (mm/min) limit over one minute interval where, *L* = span length of the beam (mm), and *d* = effective depth of the beam (mm). The time at which any of the applicable failure limit states is exceeded is taken as fire resistance of the flexural member.

### 5.3 Material properties

The temperature dependent thermal, mechanical, and deformation properties, considered in heat transfer and sectional analysis, are defined using the relations specified in ASCE manual [16] and Eurocode-2 [17] for concrete and reinforcing steel rebars, respectively. The relations proposed by Bai et al. [18] and the test data reported by Bhatt [11,12] dependent thermal properties of different fire insulation materials. The degradation of strength properties of FRP with temperature is defined using the relations proposed by Bisby et al. [19], whereas the temperature dependent bond-slip relations proposed by Dai et al. [20] are used to define the FRP-concrete interfacial bond behavior.

### 5.4 Model validation

The above-described model is applied to undertake fire resistance analysis on the fire tested beams TB1 and TB2, and the predicted response parameters are compared with the measured values to establish the validity of the model. The geometric configuration and material properties of the strengthened T-beams are identical to those in the fire test. For the analysis, the length of the beams is discretized into 20 segments. Further, the concrete, CFRP, and insulation in the cross-section of each of these 20 segments is discretized into quadrilateral elements of size 20 mm × 20 mm, *t_frp_* × 10 mm, and 5 mm × 5 mm elements, respectively, where *t_frp_* is the thickness of CFRP sheet. Both the beams have structural loading and boundary conditions similar to those in test. Moreover, the furnace temperature measured in tests is applied to the bottom and sides of the beams to simulate the thermal loading on the beams similar to that in test. The detailed input files for thermal and structural analysis of beams TB1 and TB2 are provided in the dataset repository [2].

To validate the thermal response, the predicted and measured temperatures at the FRP-concrete interface and mid steel rebar of the beams are compared in Fig. 3 (a and b), respectively. The figures clearly indicate that the predicted temperature rise at these locations agrees with the measured temperature rise, with minor discrepancies. For instance, the water evaporation plateau observed in the fire test is not predicted by the model as the effect of water vaporization is not considered in the model. Further, the sharp increase in the FRP-concrete interface temperature is not predicted by the model as the insulation delamination is also explicitly accounted for in model. Overall, the predicted temperature rise trends match well with the measured trends indicating that the model can predict thermal response with sufficient accuracy.

To validate the structural response, the predicted and measured mid-span deflection of the beams is compared in Fig. 3(c). It can be seen from the figure that the predicted deflection compares well with the measured values with slightly higher prediction in the later stages of fire exposure. However, the overall trends of the measured and predicted deflection are almost similar. Thus, the model can predict the fire response of FRP-strengthened flexural members with sufficient accuracy. Additional details pertaining to the numerical procedure, material properties, and failure criteria implemented in the model can be found in [1], while the details pertaining to the validation or other outputs generated from the model can be found in [11,12]. The validated numerical model was further applied to generate Dataset-N (spreadsheet “02_NumericalModelData”) on fire resistance analysis of FRP-strengthened concrete beams with different geometrical configuration, material properties, and applied structural loading.

## Limitations

The database presented in the paper comprises of more than 21,000 datapoints with varying values for different parameters influencing the fire performance of FRP-strengthened RC beams. However, only five different types of insulation materials were considered for generating the numerical data. Similarly, all the beams provided in the database were tested or analyzed under standard fire exposure scenario. Therefore, the database cannot be utilized for conducting statistical or machine learning analysis for FRP-strengthened RC beams exposed to real fire scenario. These are some of the major limitations associated with the database presented in the paper.

## Publication Statements

### Ethics statements

The authors would like to confirm that they have read and followed the ethical requirements for publication in *Data in Brief*. The authors would also like to confirm that no human subjects, animal experiments, and social media platforms were used for developing the data presented in the paper.

## CRediT authorship contribution statement

**P.P. Bhatt:** Conceptualization, Methodology, Formal analysis, Data curation, Writing – original draft. **V.K.R. Kodur:** Methodology, Writing – review & editing, Supervision. **M.Z. Naser:** Methodology, Writing – review & editing.

## Data Availability

Fire Resistance of FRP-Strengthened Beams (Original data) (Mendeley Data)
